# Prodromal research: Public health initiatives for prevention of schizophrenia

**DOI:** 10.4103/0019-5545.58889

**Published:** 2010

**Authors:** Amresh Shrivastava

**Affiliations:** Mental Health Foundation of India, Prerana Charitable Trust, 209 Shivkripa Complex, Gokhale Road, Thane, Mumbai - 400 602; Maharashtra, India, Early Intervention of Psychosis Program, Regional Mental Health Care. St. Thomas, The University of Western Ontario, and Associate Scientist, Lawson Health Research Institute, London, Ontario, Canada

Scientific community is excited with significant research data gathered during past 10 years to identify and predict the vulnerability of individuals to develop psychosis. Researchers in prodromal psychosis are forcefully arguing for inclusion of ‘risk syndrome of psychosis’ as a diagnostic category in DSM V. This has generated an interesting debate.[1] Major research achievements in this field have indicated that it is possible to identify candidates who might develop psychosis. It is also known that delay in treatment compromises the outcome, as most of the functional damage is caused much before the florid symptoms appear. Psychosis being a neurotoxic state; once the ‘critical period’ for intervention is missed, the treatment outcome shall remain poor. Though early-intervention initiative has found political ground in most of the developed countries, the patient in developing world continues to struggle with the system, management, finance and manpower to translate this research into practice. While academia is debating whether identifying risk syndrome of psychosis is worthy of a diagnostic category or not, we forget that it is irrelevant for the patient.

At this juncture, the basic principle of medicine needs to lead the argument in psychiatry. Even if there is slightest suspicion of a disabling condition like schizophrenia, all possible, care, caution and compassion must be in operation. Studies indicate that approximately 80-85% ‘at-risk’ individuals report experiencing subsyndromal symptoms for a period lasting several months to several years prior to the onset of the illness.[2] Such symptoms include impaired perception, thought processes, subjective cognitive functions and mood symptoms. Much of the functional decline associated with schizophrenia occurs during this prodromal phase.[3,4] Researchers consider that if sensitive and specific diagnostic criteria for prodrome can be developed, it may be possible to address the functional impairments associated with the psychotic prodrome. There is a hope that ‘forward movement in recognizing’ predictive factors, biological and clinical marker for endophenotypes may provide a framework for prevention. It is therefore very significant advancement in the pathway of schizophrenia research. This paper examines arguments for re-visiting traditional concept of prevention with modern research findings. The concept and phenomenology of prodrome is briefly discussed to assess whether these scientific achievements can be tailored into practice in the community. The concept of early intervention in psychiatric disorders is not a new one. Early identification has been a classical public health teaching. The challenge has been in developing the methodology to practice the scientific ideology. One way to deal with these challenges has been to develop new scientific argument for the earliest intervention. It is a paradox that the acceptance of early-intervention paradigm as the ‘correct’ strategy should have to be proven through extensive research. How ethical is it to allow a young person suffering from psychosis to go undiagnosed and untreated, and for how long? Despite the important and impressive results from early-intervention services around the globe, the socio-economic divide and diversity continues to be the main hurdle in success of pragmatic approaches.

A prodrome has been defined as ‘an early or premonitory manifestation of impending disease, before specific symptoms begin,’ and a prodrome of schizophrenia has been described since the time of Bleuler.[5] A prodrome for schizophrenia can be identified only in retrospect, after the disease has been diagnosed, when the opportunity for preventing onset is past. ‘The prodromal risk syndrome for psychosis’ is a term applied here to the condition studied using prospective methods by researchers interested in prevention. It suggests that syndrome carrying a substantial risk of progression to full-blown psychosis in the near future is characterized by evolving attenuated positive symptoms, negative symptoms and functional impairment.[6] Findings from at-risk research studies in a number of countries document the validity of pre-psychosis risk detection by observing non-trivial rates of conversion to psychosis in a short time period.

Data from controlled studies suggest that clinical intervention may be effective in delaying or preventing exacerbation into psychosis, but evidence to date is very weak. Screening for risk of psychosis is certainly controversial.[7] Nonetheless, there is a clinical need for methods of identification and intervention preceeding fully manifested psychosis. Predictive power for progression along this path is enhanced by family history of psychotic illness, but the gain in predictive accuracy is offset by a sharply reduced sensitivity. Success with these criteria suggests a compelling need of a diagnostic class, a view supported by the help-seeking status of patients and families in these studies.[5] Research data is neither sufficient nor irrefutable to consider this as a diagnosis by itself. Diagnosis and quantification of ‘at-risk’ has been a challenge thus far. Significant work has been done in developing definitions for validation and instruments for quantification.

Instruments have been developed to assess the cluster of subthreshold symptoms. It will be imperative to determine if early intervention, perhaps years in advance of psychosis, is effective and favorably alters the future course of illness. The critical period hypothesis proposes that the early phase of psychosis, including any period of initially untreated psychosis, is a ‘critical period’ during which Symptomatic and psychosocial deterioration progresses rapidly.[8] Afterwards, progression of morbidity slows or stops, and the level of disability sustained, or recovery attained, by the end of the critical period endures into the long term.

The finding of ‘Duration of Untreated Psychosis’ (DUP) has acquired a position of being an independent marker associated with the outcome. However the consistency of association seen in various studies has been poor. People with poor premorbid adjustment are known to have a poor outcome and could be reluctant to make contact with the services. Despite the promising finding of DUP and its association with outcome, it would be naïve at this stage to predict that reducing DUP will improve outcome.[9] The initiative of early intervention and first-episode psychosis has provided a new insight into the aetiopathological process of the illness. Studies have shown reduced relapse rate, better functions and enhanced patient's satisfaction if intervention is done in early phase of illness.[10,11]

However, despite the blossoming of early-intervention services, there is continuing disagreement over whether there is a real association between DUP and outcome. Several conflicting evidence have been reported.[12] The strength of association between DUP and outcome has been found to be only ‘moderately strong’ based upon the available data, accounting for approximately 13% of variance or 1 in 3 to 1 in 4 of those who did not achieve remission.[13] Functional recovery is a collective impact of improvement on several parameters representing functions in everyday life.[14] Most likely several other factors together with short DUP may determine a ‘good outcome’.[15,16] A number of markers for endophenotypes of schizophrenia either lack specificity or may be changing over the initial phases of illness indicating that they do not represent stable or even enduring trait markers. The most promising marker such as executing function and more direct measures of frontal lobe integrity relate to frontal and perhaps temporal cortices, which are the brain regions that are changing dynamically during adolescence and early adulthood.[17] Lack of specificity of these markers continues to elude researcher in the field. List of common prodromal symptoms includes reduced concentration and attention, reduced drive and motivation, depression, sleep disturbance, anxiety, social withdrawal, suspiciousness, deterioration in role functioning and irritability. Closer to onset of frank psychotic symptoms people often experience attenuated or subthreshold symptoms. Neurocognitive abnormalities are also evident in prodromal phase, and individual diagnosed of prodromal phase displays a range of abnormalities similar to those found with first-degree relatives of patients with schizophrenia. Our knowledge that many symptoms and a great deal of disability develop during the prodrome coupled with the finding of possible neurobiological and neurocognitive damage during this period has added impetus for renewed efforts for intervention at an early stage.

Research has also attempted to detect the presence of such deficits prior to the illness onset, as they represent trait markers. Some of these are working memory, immediate verbal recall deficit, rapid registration, efficient recall and olfactory identification deficit. It has also been demonstrated that these changes are similar to those occurring in the first-degree relative of schizophrenia.[18]

Based upon this understanding the staging model was developed, which is revolving around the theme of early diagnostic markers or risk factors for psychotic illness or isolated distressing and debilitating symptoms.[19] According to this concept the psychosis spans from normal state zero to severe psychosis, stage 4b, on a spectrum of psychopathology.[20]

Studies like ‘Ultra High Risk’ (UHR)or prodromal trials and the greater effectiveness and acceptability of ‘First episode psychosis’ (FEP) program provides real support for the staging model. There has also been remarkable research in substance abuse and suicidality in early psychosis.

The onset of substance abuse precedes the onset of psychosis in most individuals with comorbid substance misuse in first-episode psychosis.[21] Out of several hypotheses to explain this complex link between substances and psychosis, the most convincing is the one that argues for common risk factors for psychosis and substance abuse due to dopamine as the final common pathway neurobiologically.[22]

Understanding the ‘process of suicidality’ in psychosis both during psychotic state and non-psychotic state and recognizing the valid predictors has been one of the main challenges in the research. Suicide in prodromal phase is under-recognized and poorly studied. It is true that hardly a few of these individuals seek professional help. There is a greater awareness that the early years of psychosis represent a period of highest risk for suicide and that early-intervention strategies may reduce the risk. Though this would be most desirable, there is a lot of skepticism in this hypothesis.

Historically, lessons of early identification have been in the forefront of community and preventive approach of psychiatry. The scientific evidence that delay is disastrous for outcome is far clearer now and generates hope in millions who become ‘non-exist’. The findings of the prodromal as well as early psychosis research go much beyond the context it refers to. It has many advantageous clinical and social implications.

The first implication is the enhanced confidence of the clinician and matter of hope for the consumer. The scientific evidence is enough for proactive practice of psychiatry. The message can be very effectively taken to the community with awareness programs saying that it is possible to change and improve the situation. Program evaluation shows encouraging results and provides further argument for governments to continue to invest in early intervention. The pharmaco-economic evaluations are also robust. A chain reaction of identification and intervention provides new kindling for preventive efforts.

The second important implication is the indirect benefit to a large number of patients who have excellent opportunity for timely treatment due to new early-intervention programs. Governments have come forward with funding and resources and in many countries early intervention is available not only for psychosis programs, but also across the psychiatric diagnosis. It is now a political agenda. Community leaders encourage a mental health program in their catchment.

The third and equally important factor is the impact on awareness and stigma. It is a new wave of ‘information with evidence’. The general excitement, economics, politics and activism have made definite impact of mental health on public at large. Internet as medium of communication has played an important role in this initiative. The content and level of discussion is now enormously enriched between patients, relatives and treatment team.

The fourth and the most significant factor is the strategic development of ‘Health Service Research’ model. For the first time the experiment to establish such a model of research in mental health has been truly successful. It was attempted in addictions, dual disorders and several other subjects, but it did not come out as impressive as ‘early intervention’ has. There is an ongoing cycle of ‘research-to-clinic-to-research’, which substantiates that quality. Researchas a clinical science, has several stakeholders, and it is possible to involve all of them and innovate. The program provides true examples for partnership in care. The lesson learnt from prodromal and early intervention research is encouraging. It demonstrates that clinics are the laboratories for psychiatric research and how psychiatric research advances clinical care. In this way the consensus statement for prevention of schizophrenia gets further strength.[23]

The power now lies in the hands of the consumers, policy development professionals, Governments and economists to demonstrate their will and wisdom in developing the services across regions and cultures that have been denied because of ‘lack of evidence’.
